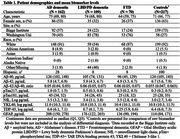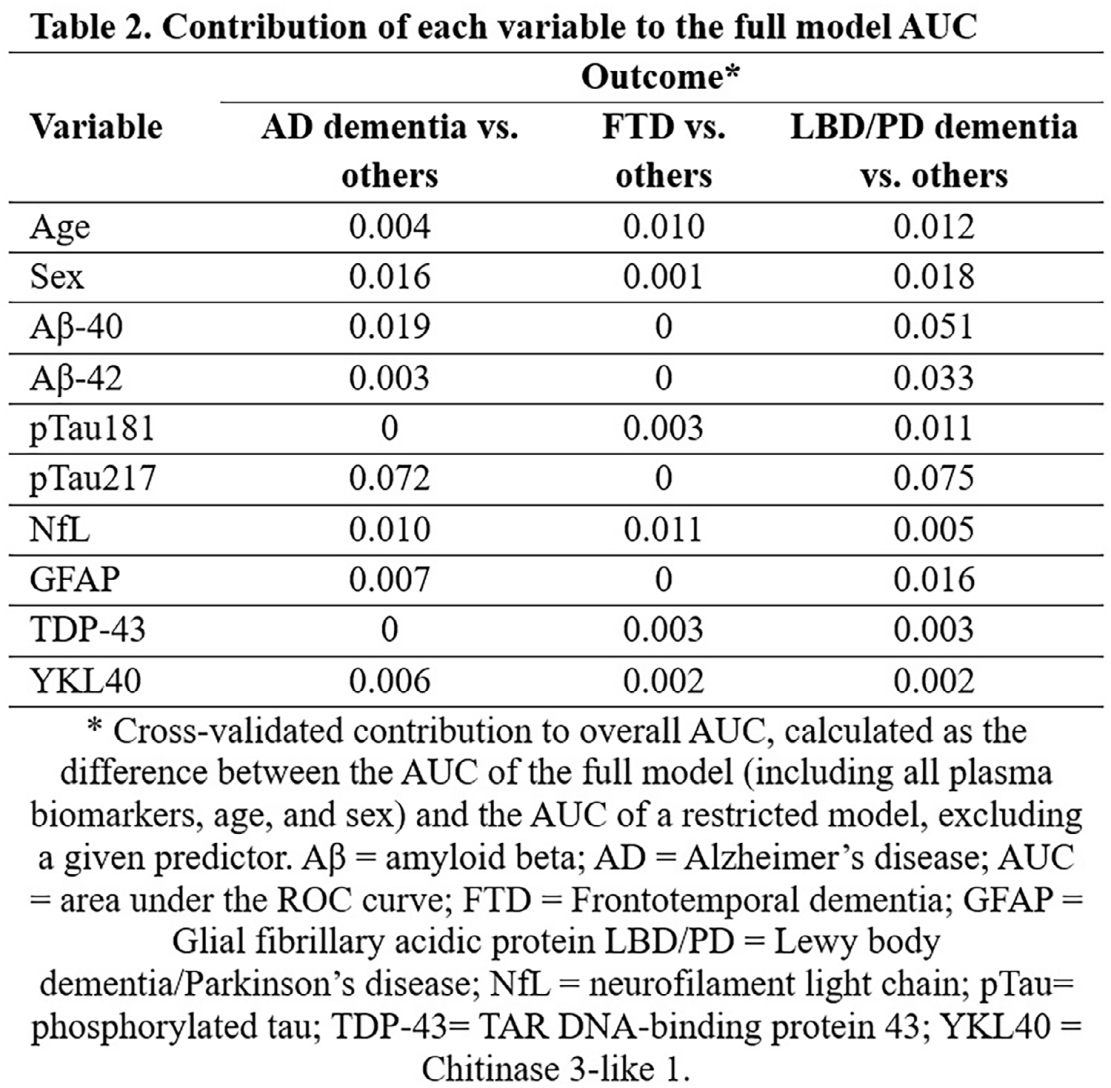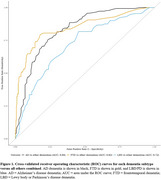# Plasma biomarkers for the differential diagnosis of dementia subtypes in clinic

**DOI:** 10.1002/alz70856_104637

**Published:** 2025-12-26

**Authors:** Stephanie Yiallourou, Tiffany F. Kautz, Julie Parker‐Garza, Lachlan Cribb, Pat Kohlfeld, A. Campbell Sullivan, Ashley LaRoche, Jeremy A. Tanner, Bernard Fongang, Claudia L Satizabal, Crystal Wiedner, Jayandra Jung Himali, Carlos Cruchaga, Mitzi M. Gonzales, Sudha Seshadri, Matthew P. Pase

**Affiliations:** ^1^ School of Psychological Sciences & Turner Institute for Brain and Mental Health, Monash University, Melbourne, VIC, Australia; ^2^ Glenn Biggs Institute for Alzheimer's and Neurodegenerative Diseases, University of Texas Health Science Center, San Antonio, TX, USA; ^3^ Glenn Biggs Institute for Alzheimer's & Neurodegenerative Diseases, UT Health San Antonio, San Antonio, TX, USA; ^4^ Monash University, Melbourne, VIC, Australia; ^5^ NeuroGenomics and Informatics Center, Washington University School of Medicine, St. Louis, MO, USA; ^6^ Glenn Biggs Institute for Alzheimer's & Neurodegenerative Diseases, University of Texas Health Science Center, San Antonio, TX, USA; ^7^ Glenn Biggs Institute for Alzheimer's and Neurodegenerative Diseases, University of Texas Health Science Center, San Antonio, TX, USA; ^8^ Glenn Biggs Institute for Alzheimer's & Neurodegenerative Diseases, University of Texas Health Science Center, San Antonio, TX, USA; ^9^ Glenn Biggs Institute for Alzheimer's & Neurodegenerative Diseases, The University of Texas Health Science Center at San Antonio, San Antonio, TX, USA; ^10^ Glenn Biggs Institute for Alzheimer's & Neurodegenerative Diseases, University of Texas Health San Antonio, San Antonio, TX, USA; ^11^ Department of Genetics, Washington University School of Medicine, St Louis, MO, USA; ^12^ The Charles F. and Joanne Knight Alzheimer Disease Research Center, St Louis, MO, USA; ^13^ Cedars‐Sinai Medical Center, Los Angeles, CA, USA; ^14^ Monash University, Clayton, VIC, Australia

## Abstract

**Background:**

Plasma biomarkers demonstrate strong performance in detecting Alzheimer's disease (AD). However, their role in the differential diagnosis of dementia subtypes remains underexplored, particularly in the early clinical stages of AD, frontotemporal dementia (FTD), and Lewy body/Parkinson's disease (LBD/PD) dementia. This study aimed to evaluate the performance of plasma biomarkers in differentiating these dementia subtypes using a machine‐learning approach.

**Methods:**

Patients with a clinical diagnosis of early AD, FTD, and LBD/PD dementia were recruited from the Knight ADRC at Washington University and the Biggs Institute for Alzheimer's and Neurodegenerative Diseases. Plasma samples were analyzed for markers of neuropathology, neuronal injury, and neuroinflammation, including Aβ40, Aβ42, pTau181, pTau217, TDP‐43, NfL, GFAP, and YKL40. All biomarkers were measured centrally (Biggs Institute) on the Simoa platform, except YKL40 (MSD). Dementia subtype classification models (e.g., AD vs. others) were developed using the SuperLearner algorithm, which combines multiple machine learning models into an optimally weighted prediction. Models included age, sex, and all plasma biomarkers. Performance was assessed using cross‐validated area under the receiver operating characteristic curve (AUC). The contribution of each biomarker to the AUC was calculated by comparing the full model to a restricted model excluding that biomarker.

**Results:**

Biomarker data was obtained on 162 patients with AD, 70 with FTD, and 109 with LBD/PD (Table 1). Models combining all biomarkers showed excellent discrimination of AD dementia (AUC=0.84) and FTD (AUC=0.85) from other dementias, and moderate discrimination for LBD/PD dementia (AUC=0.72; Figure 1). Among individual biomarker contributions (Table 2), pTau217 was the strongest predictor for AD differentiation. The low individual contributions for FTD suggest that FTD differentiation was driven by a combination of biomarkers, with NfL showing the highest individual contribution. For LBD/PD, Aβ40 and pTau217 contributed the most, though model performance was moderate.

**Conclusion:**

pTau217 emerged as the key biomarker for AD differentiation. The smaller independent contributions of individual biomarkers to FTD classification suggest that predictive information is shared across multiple markers, with NfL having the highest contribution. Plasma biomarkers show strong potential for aiding early, non‐invasive differential diagnosis of AD and FTD. Further optimization is needed for LBD/PD classification.